# Population productivity of shovelnose rays: Inferring the potential for recovery

**DOI:** 10.1371/journal.pone.0225183

**Published:** 2019-11-21

**Authors:** Brooke M. D’Alberto, John K. Carlson, Sebastián A. Pardo, Colin A. Simpfendorfer

**Affiliations:** 1 Centre for Sustainable Tropical Fisheries and Aquaculture & College of Science and Engineering, James Cook University, Townsville, Queensland, Australia; 2 CSIRO Oceans and Atmosphere, Hobart, Tasmania, Australia; 3 NOAA/National Marine Fisheries Service–Southeast Fisheries Science Center, Panama City, FL, United States of America; 4 Biology Department, Dalhousie University, Halifax, NS, Canada; Australian Bureau of Agricultural and Resource Economics and Sciences, AUSTRALIA

## Abstract

There is recent evidence of widespread declines of shovelnose ray populations (Order Rhinopristiformes) in heavily fished regions. These declines, which are likely driven by high demand for their fins in Asian markets, raises concern about their risk of over-exploitation and extinction. Using life-history theory and incorporating uncertainty into a modified Euler-Lotka model, the maximum intrinsic rates of population increase (*r_max_*) were estimated for nine species from four families of Rhinopristiformes, using four different natural mortality estimators. Estimates of mean *r_max_*, across the different natural mortality methods, varied from 0.03 to 0.59 year^-1^ among the nine species, but generally increased with increasing maximum size. Comparing these estimates to *r_max_* values for other species of chondrichthyans, the species *Rhynchobatus australiae*, *Glaucostegus typus*, and *Glaucostegus cemiculus* were relatively productive, while most species from Rhinobatidae and Trygonorrhinidae had relatively low *r_max_* values. If the demand for their high-value products can be addressed then population recovery for some species is likely possible, but will vary depending on the species.

## Introduction

An estimated 25% of chondrichthyan (sharks, rays and chimeras) populations have an elevated risk of extinction [1], raising significant ecological and conservation concerns [2–4]. Chondrichthyans, generally have low biological productivity (slow growth, late maturity, few offspring, and long generational times), which limits their ability to recover from population declines [5, 6]. Declines of chondrichthyan populations are typically the result of the rapid expansion of fisheries [7–9] and the globalisation of trade [10, 11], and can be exacerbated by habitat degredation [12]. Compared to other chondrichthyans, larger elasmobranchs (sharks and rays, Subclass Elasmobranchii) have some of the lowest intrinsic rates of population increase [13, 14], and as a result are unlikely to sustain high levels of fishing pressure before population collapse [15–18].

The order Rhinopristiformes is considered one of the most threatened orders of marine fish [1, 19], and comprises five families: sawfish (Pristidae), giant guitarfish (Glaucostegidae), wedgefish (Rhinidae), guitarfish (Rhinobatidae) and banjo rays (Trygonorrhinidae) (Table 1) [19, 20]. These large rays are strongly associated with soft-bottom habitats in shallow (<100 m) tropical and temperate coastal waters [21–23], resulting in high exposure to intensive and expanding fisheries [24]. These coastal habitats are under threat from anthropogenic influences, which is also a significant threat for these rays [25, 26]. They are very susceptible to overexploitation as a result of their large body size [1], high catchability by multiple gear types [27], and use of inshore habitat in some of the world’s most heavily fished coastal regions [28–30].

**Table 1:** The nine species of shovelnose rays in this study. Listed is their threat status according the International Union of Conservation of Nature’s (IUCN) Red List of Threatened Species, and whether the species are listed on the appendixes of CITES, and/or CMS, and the CMS Sharks MOU (MOU). IUCN categories are CR, Critically Endangered; EN, Endangered; VU, Vulnerable; LC, Least Concern; DD, Data Deficient.

Family	Species	IUCN	Year	CITES	Year	CMS	Year
Rhinidae	*Rhynchobatus australiae*	CR	2019	Appendix II	2019	Appendix II/ MOU Annex 1	2017 2018
Glaucostegidae	*Glaucostegus cemiculus*	CR	2019	Appendix II	2019	-	-
	*Glaucostegus typus*	CR	2019	Appendix II	2019	-	-
Rhinobatidae	*Acroteriobatus annulatus*	LC	2006	-	-	-	-
	*Pseudobatos horkelii*	CR	2007	-	-	-	-
	*Pseudobatos productus*	NT	2014	-	-	-	-
	*Rhinobatos rhinobatos*	EN	2007	-	-	Appendix II/ MOU Annex 1	2017 2018
Trygonorrhinidae	*Zapteryx brevirostris*	VU	2006	-	-	-	-
	*Zapteryx exasperata*	DD	2015	-	-	-	-

There is increasing evidence of historical and contemporary declines in landings and catch rates for wedgefishes, giant guitarfishes, guitarfishes and banjo rays (herein collectively referred to as shovelnose rays), of up to 80% throughout most of their ranges [24], including Indonesia [31], South Africa [32], Madagascar [33], Mozambique [34], Tanzania [35], Arabian Seas and surrounding region [19, 36], India [37] and Brazil [38]. Many species of shovelnose rays are facing a high to extremely high risk of extinction in the wild [24, 39, 40]. While there are very few directed fisheries (e.g. Indonesian tangle-net fishery [27]) for shovelnose rays, they are typically retained in commercial and artisanal fisheries as by-products for their highly valued fins and good quality meat [24, 41, 42]. Wedgefish and giant guitarfish fins are considered the highest grade fins [7, 25, 31, 43]. The reported declines of landings and catches of shovelnose rays are likely to be primarily driven by the international shark fin trade as they are prevalent in fin trading hubs such as Hong Kong [44] and Singapore [45, 46]. There is considerable concern that shovelnose rays, in particular wedgefishes and giant guitarfishes, are following a similar pattern of global decline as the sawfishes [19, 24]. All five species of sawfish declined rapidly over 30 years throughout their range, driven by unregulated fisheries, the interational fin trade, and delayed scientific attention [47–50]. Yet despite a global conservation strategy [25], restriction of international trade (i.e. listing on Convention on International Trade in Endangered Species of Wild Fauna and Flora [CITES] Appendix I), and evidence that some species of sawfish have the ability to recover from fishing pressure [51], the recovery of the populations is projected to take at least several decades. Precautionary management and conservation of shovelnose rays is therefore vital to maintain their populations.

Currently, fisheries for shovelnose rays are not regulated through national or regional species-specific fishing regulations. The magnitude of declines in landings in heavily fished regions, and the subsequent conservation issues have attracted the focus of major international management conventions and agencies, such as the Convention on the Conservation of Migratory Species of Wild Animals (CMS; *Rhynchobatus australiae* and *Rhinobatos rhinobatos* listed on the Appendix II) [52], the non-binding CMS Memorandum of Understanding on the Conservation of Migratory Sharks (CMS Sharks MOU; *R*. *australiae*, *Rhynchobatus djiddensis*, *Rhynchobatus laevis*, and *R*. *rhinobatos* listed on Annex 1) [53], and CITES (families Rhinidae and Glaucostegidae listed on Appendix II) [54]. For CITES Appendix II listed species, the international trade of wild specimens must be legal and sustainable, which is dependent on provisions such as the export is not detrimental to wild populations (through a positive non-detriment finding, NDF), the specimens are legally sourced, and shipments are accompanied by export, import or re-export permits [55]. While the CMS Appendix II listing acts as a framework for the Range States (any Party [nation] that exercises jurisdiction over any part of the range of that migratory species) of the migratory species that have unfavourable conservation status, and requires international agreements [56]. These international agreements provide a global platform and legal foundation for the conservation and sustainable use of internationally traded species (CITES), and migratory species and their habitat (CMS) [55]. Given the global concerns for this group of species and the importance of trade in their high-value fins, the use of international trade regulations through CITES listings may help achieve positive conservation outcomes [24, 55, 57]. Successful recovery of populations will require significant measures across local, regional and global scales [57]. However, management and conservation efforts can be hampered by the lack of understanding of life-history (e.g. age, growth and maturity), demographic information, and recovery rates.

Understanding the ability of species to recover from declines following implementation of management measures is important for rebuilding depleted populations. This can be approximated through measuring the species’ population productivity using various demographic techniques such as rebound potential models [58–60], age or stage structured life-history tables and matrix models [61, 62], and demographic invariant methods [63, 64]. These demographic techniques utilise the known relationships between life-history traits and demography, known as the Beverton-Holt dimensionless ratios [65] that can be used to infer a species’ life-history traits based on known parameters [66–68]. One commonly used metric of productivity is the maximum intrinsic rate of population increase *r_max_*, which reflects the theoretical maximum growth rate of depleted populations in the absence of density dependent regulation [69]. This method can help to infer and understand a species ability to recover from population declines, and provide the demographic basis for evaluating the sustainability of fisheries [70] and international trade, particularly for poorly monitored species with limited available life-history information [71, 72]. The maximum intrinsic population rate of population increase has previously been estimated for *Pseudobatos horkelii* and *Pseudobatos productus* as a part of multi-species comparison [72, 73], however there has not been a comprehensive analysis on the population productivity for shovelnose rays.

The aim of this paper was to use life-history data and theory to estimate the population productivity for shovelnose rays. The focal families studied were wedgefishes, giant guitarfishes, guitarfishes and banjo rays, while the sawfishes were excluded as they have been previously assessed in detail [50]. The population productivity of these rays was compared to available productivity estimates of 106 other shark and ray species.

## Materials and methods

### Life-history data collection

A literature search was conducted for all species from the four families of shovelnose rays to provide data for estimation of population productivity. Life-history information required for analyses consisted of age at maturity (*α_mat_*, range of years), maximum age (*α_max_*, in years), range of litter size (in number of female pups), sex ratio, breeding intervals (*i*, years), and von Bertalanffy growth coefficient (*k*, year^-1^). Out of the four families, with a total of 57 species, only nine species had enough published life-history information to estimate *r_max_* (Table 1).

Growth coefficient data for *R*. *australiae* were reported as *Rhynchobatus spp*. by White *et al*. [74] as results from the species complex including *R*. *australiae*, *Rhynchobatus palpebratus* and *Rhynchobatus laevis* along the eastern coast of Australia. Recent taxonomic revision has resolved this species complex in this area, with *R*. *laevis* primarily found in the Indian Ocean and Indo-West Pacific Ocean [75], and further examination of data, including genetic analysis, associated with specimens examined by White *et al*. [74] have demonstrated they were primarily *R*. *australiae*. The three parameter von Bertalanffy growth rate was estimated for *R*. *australiae* and *G*. *typus* using extracted length at age data from White *et al*. [74] (see S1 Appendix for methods). This was done as White *et al*. [74] only reported the two parameter von Bertalanffy growth rate for these two species, where the size at birth parameter (*L_0_*) is fixed to an empirically estimated length [76] and substantially biases the growth estimates [77, 78]. For *R*. *australiae*, *G*. *typus* and *Z*. *brevirostris* the age at maturity was back-calculated using:

$${Age}_{x}=\frac{\left(ln(L_{\infty }-{TL}_{x})-ln(L_{\infty })-(k*t_{0})\right)}{-k}$$

where *Age*_*x*_ is age at time x, *TL*_*x*_ is total length (cm TL) at time x, *L*_∞_ is the asymptotic length (cm TL), *t*_0_ is the length at time zero, and *k* is the von Bertalanffy growth coefficient. For *R*. *australiae*, the age at maturity was back-calculated using the von Bertalanffy parameters reported for *Rhynchobatus spp*. [74] and the size at maturity of 150 cm TL from *Rhynchobatus djiddensis* [75]. The age at maturity for *Glaucostegus typus* was estimated using the estimated size at maturity [75] and growth coefficient [74]. There is no reported litter size for *G*. *typus*, thus we assumed it had the same litter size and breeding interval as *Glaucostegus cemiculus* to calculate annual reproductive output. For *R*. *australiae*, *Acroteriobatus annulatus*, *Zapteryx exasperata* and *Z*. *brevirostris*, the breeding interval was assumed to be one year, as there was no information available (Table 2).

**Table 2:** Life-history values and sources used to estimate *r*_*max*_ for the nine species of shovelnose rays. Including the maximum size (L_max_ in centimetres total length, cm TL), lower, upper and mean (standard deviation, S.D.) values of the age at maturity (*α*_*mat*_, years), lower and upper values for litter size, breeding interval (*i*, years), lower and upper annual reproductive output of females (*b*), lower and upper values for von Bertalanffy growth coefficient (*k*, year^-1^), the observed, and lower (*T*_*lower*_) and upper (*T*_*upper*_) and mean (S.D.) values of theoretical maximum age (*α*_*max*_, years). See Table 1 in S1 Appendix for re-estimated *k* results for *R*. *australiae* and *G*. *typus*.

	L_max_	α_mat_				litter size		i	*b*		*k*		α_max_					
Species	(cm TL)	lower	upper	mean	± S.D.	lower	upper	(years)	lower	upper	lower	upper	Observed	T_lower_	T_upper_	mean	± S.D.	References
*Rhynchobatus australiae*	300	3.00	6.00	4.50	0.450	7	19	1	3.5	9.5	0.083	0.400	12.0	11.3	22.3	16.78	0.76	[74, 75]
*Glaucostegus cemiculus*	290	2.89	6.50	4.70	0.680	5	24	1	2.5	12	0.200	0.275	14.0	13.9	16.1	14.67	0.50	[75, 79–82]
*Glaucostegus typus*	270	6.50	8.00	7.25	0.245	5	24	1	2.5	12	0.040	0.150	19.0	18.1	27.4	22.74	0.16	[74, 75, 83]
*Acroteriobatus annulatus*	140	2.30	2.80	2.55	0.080	2	10	1	1.0	5.0	0.240	0.240	7.00	14.8	14.8	12.23	1.30	[75, 84]
*Pseudobatos horkelii*	140	7.00	9.00	8.00	0.300	4	12	1	2.0	6.0	0.194	0.194	28.0	16.3	16.3	22.17	1.86	[75, 85]
*Pseudobatos productus*	170	7.00	8.40	7.70	0.200	1	10	1	0.5	5.0	0.016	0.240	33.8	14.8	33.8	33.80	3.50	[75, 86–88]
*Rhinobatos rhinobatos*	185	2.20	4.10	3.15	0.350	1	14	1	0.5	7.0	0.134	0.310	18.9	13.1	18.9	18.92	1.00	[75, 89–94]
*Zapteryx brevirostris*	66.0	7.71	11.5	9.61	0.700	1	8	1	0.5	4.0	0.110	0.130	10.0	19.1	20.3	16.48	1.55	[75, 95–98]
*Zapteryx exasperata*	103	5.41	9.65	7.53	0.800	2	13	1	1.0	6.5	0.144	0.174	22.6	17.1	18.4	19.85	0.80	[75, 99–102]

### Estimation of maximum intrinsic population growth rate, r_max_

Maximum intrinsic rate of population increase was estimated using an unstructured derivation of the Euler-Lotka model. This model accounts for juvenile survivorship that depends on age at maturity and species-specific natural mortality, and incorporates uncertainty within the parameters through Monte Carlo simulation [73, 103]. Requirements of this model are estimates of three biological parameters: annual reproductive output, age at maturity, and natural mortality. This model is founded on the principle that a breeding female only has to produce one mature female in her lifetime to ensure a stable population [104–107]:

$$l_{\alpha_{mat}}b=e^{r_{max}\alpha_{mat}}-e^{-M}{\left(e^{r_{max}}\right)}^{\alpha_{mat}-1}$$

where $l_{\alpha_{mat}}$ is survival to maturity in the absence of fishing and is calculated as $l_{\alpha_{mat}}={\left(e^{-M}\right)}^{\alpha_{mat}}$, *b* is the annual rate of production of females, *α*_*mat*_ is the age of maturity and *M* is instantaneous natural mortality. The annual reproductive output of females was calculated as *b* = 0.5*l*/*i*, where *l* is litter size (in number of males and females) and *i* is breeding interval (in years). Annual reproductive output estimates were derived from uniform distributions constrained by the minimum and maximum litter sizes published in the literature (Table 2). If the litter sex ratio was unknown, it was assumed to be 1:1. Age at maturity estimates were derived from normal distributions with means and standard deviations (S.D.) calculated from the available ages at maturity published in the literature for each species (Table 2). Normal distributions were truncated to be positive, using the standard deviations to be within “reasonable biological bounds”. The von Bertalanffy growth coefficients (*k)* for each species were derived from uniform distributions ranging between the minimum and maximum published values (Table 2). As the observed maximum age may not reflect the longevity of the species [108], the theoretical maximum age (*T_max_*) was calculating using minimum and maximum *k* reported for each species in the literature, using the following the formula [76]:

$$T_{max}=7\times ln(2/k)$$

Maximum age (*α_max_*) estimates were derived from a normal distribution using the mean and S.D., calculated from the observed maximum age reported in the literature, minimum theoretical maximum age (*T_lower_*) and maximum theoretical age (*T_upper_*). As there was no current consensus on the best indirect method to estimate the instantaneous natural mortality, it was estimated using four common methods, Jensen’s First mortality estimate [109], modified Hewitt and Hoeing estimator [110], Frisk’s estimator [66], and reciprocal of the lifespan [67] (Table 3).

**Table 3:** Natural mortality (*M*) methods used to estimate maximum intrinsic rate of population increase. Where *α*_*mat*_ is age at maturity in years, *α*_*max*_ is maximum age in years, and *k* is the von Bertalanffy growth coefficient in year^-1^.

Method	Equation	References
Jensen’s First Estimator	*M* = 1.65/*α*_*mat*_	[109]
Modified Hewitt & Hoeing Estimator	*M* = 4.22/*α*_*max*_	[110]
Frisk’s Estimator	*M* = 0.4/*k*	[66]
Reciprocal of lifespan	*M* = 1/(*α*_*mat*_+*α*_*max*_/2)	[73]

Monte Carlo simulation was used to account for uncertainty of input parameters. The annual reproductive output and age at maturity were highly uncertain parameters, while the natural mortality was estimated indirectly, which can result in additional uncertainty [13]. Model parameters were drawn from their respective distributions iteratively 20,000 times [71]. To incorporate uncertainty into *M*, for each iteration the values for *α_mat_*, *α_max_* and *k* were drawn from their respective distributions, and used to estimate natural mortality for the four natural mortality estimators, which in turn is required to estimate *r_max_* [71]. In each iteration, the *r_max_* equation was solved using the *nlminb* optimisation function by minimising the sum of squared differences. This range of *r_max_* values was generated to encompass the widest range of plausible life histories and should therefore include the true parameter values. Median and mean *r_max_* values and standard deviation were calculated.

Scenarios were investigated where uncertainty was only incorporated into a single parameter. Values of one parameter were drawn from its distribution, while the remaining parameters were set as deterministic by using the median values of their respective distributions. This was done for the age at maturity, annual reproductive output and natural mortality. The *M* value was set as deterministic in the other scenarios, even when the parameters used to estimate *M* were being drawn from distributions.

### Comparison of shovelnose ray *r_max_* estimates among chondrichthyans

Median *r_max_* of the nine shovelnose ray species were compared to all available estimates using values by Pardo *et al*. [73] to incorporate survival to maturity, including an additional 13 species (S1 Table). Following the method described above, the median *r_max_* was calculated for the additional species for which life-history information was available, including great hammerhead *Sphyrna mokarran*, smooth hammerhead *Sphyran zygaena*, common thresher shark *Alopias vulpinus*, reef manta ray *Mobula alfredi*, giant manta ray *Mobula birostris*, Chilean devilray *Mobula tarapacana*, bentfin devil *Mobula thurstoni*, blackspotted whipray *Maculabatis astra*, speckled maskray *Neotrygon picta*, narrow sawfish *Anoxypristis cuspidata*, dwarf sawfish *Pristis clavata*, smalltooh sawfish *Pristis pectinata*, and green sawfish *Pristis zijsron* (S1 Table). These species were added to increase the sample size, and to include more ray species in the analysis. The reciprocal of the lifespan natural mortality method was chosen to estimate the natural morality to compare to values generated by Pardo *et al*. [73] as that was the method used in their study. The *r_max_* estimates for *Pseudobatos horkelii* and *Pseudobatos productus* were updated with the values from this study for the comparison. The age at maturity (years), maximum age (years), growth rate (k, years^-1^) and maximum size in centimetres (cm) were plotted against the *r_max_* estimates for 115 chondrichthyan species, including the nine species of shovelnose rays. Maximum sizes were TL for all species except for Myliobatiformes, where the disc width (DW) were used [13, 72]. All models and figures were built in the R version 3.4.1 [111].

## Results

### Estimation of maximum intrinsic population growth rate, r_max_

**Fig 1:**
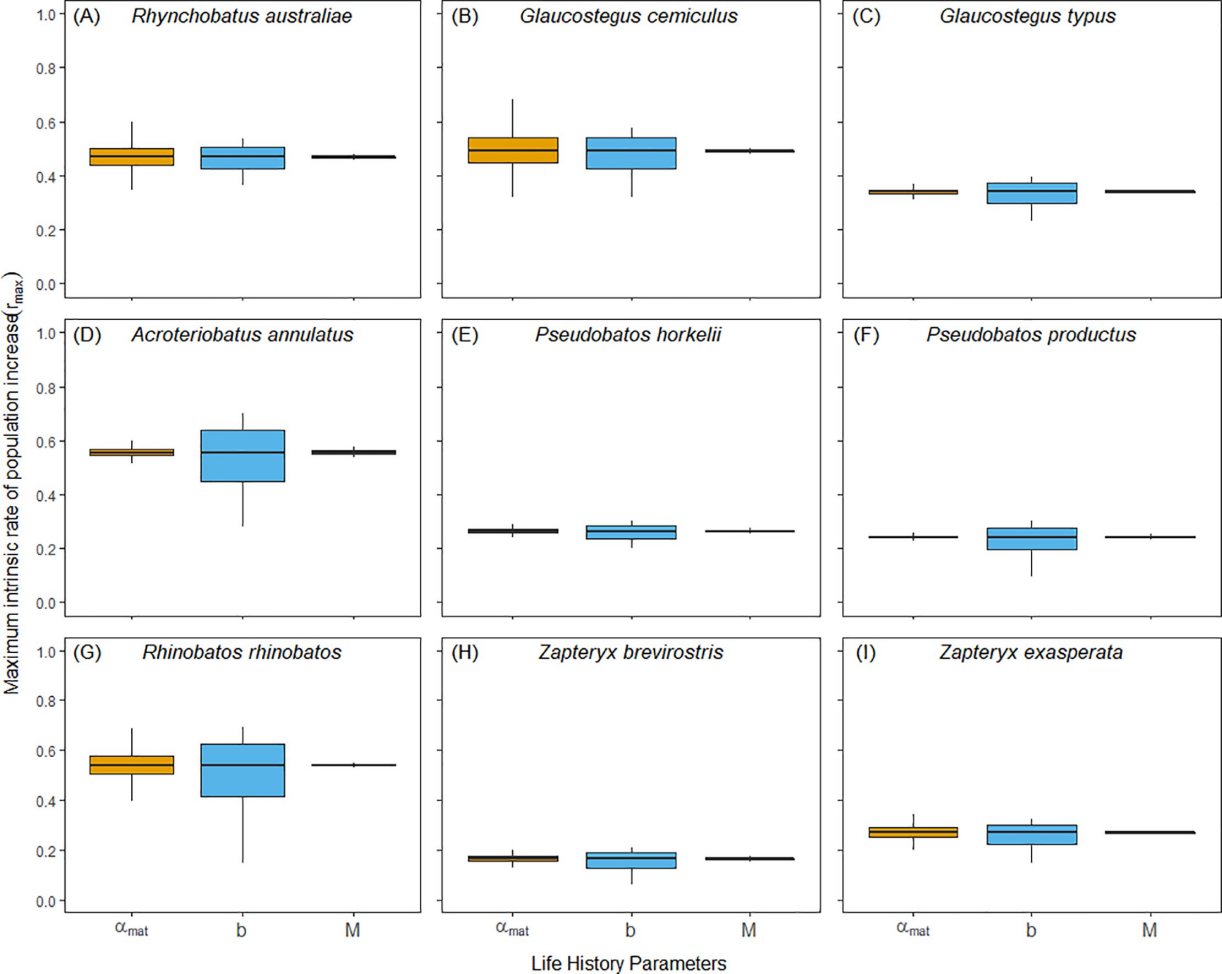
Incorporating uncertainty in the model parameters when predicting values of *r*_*max*_ (year^-1^) for nine shovelnose rays species. When including uncertainty in age at maturity (*α*_*mat*_, first/orange boxplot), annual reproductive output (*b*, middle/blue boxplot), and reciprocal of the lifespan natural mortality estimator (*M*, last/grey boxplot). Species are (A) *R*. *australiae*, (B), *G*. *cemiculus*, (C) *G*. *typus*, (D) *A*. *annulatus*, (E) *P*. *horkelii*, (F) *P*. *productus*, (G) *R*. *rhinobatos*, (H) *Z*. *brevirostris*, and (I) *Z*. *exasperata*. Boxes indicate median, 25 and 75% quantiles, whereas the lines encompass 95% of the values (2.5 and 97.5% quantiles). For plots incorporating uncertainty with other natural mortality methods, see S2 Appendix.

**Fig 2:**
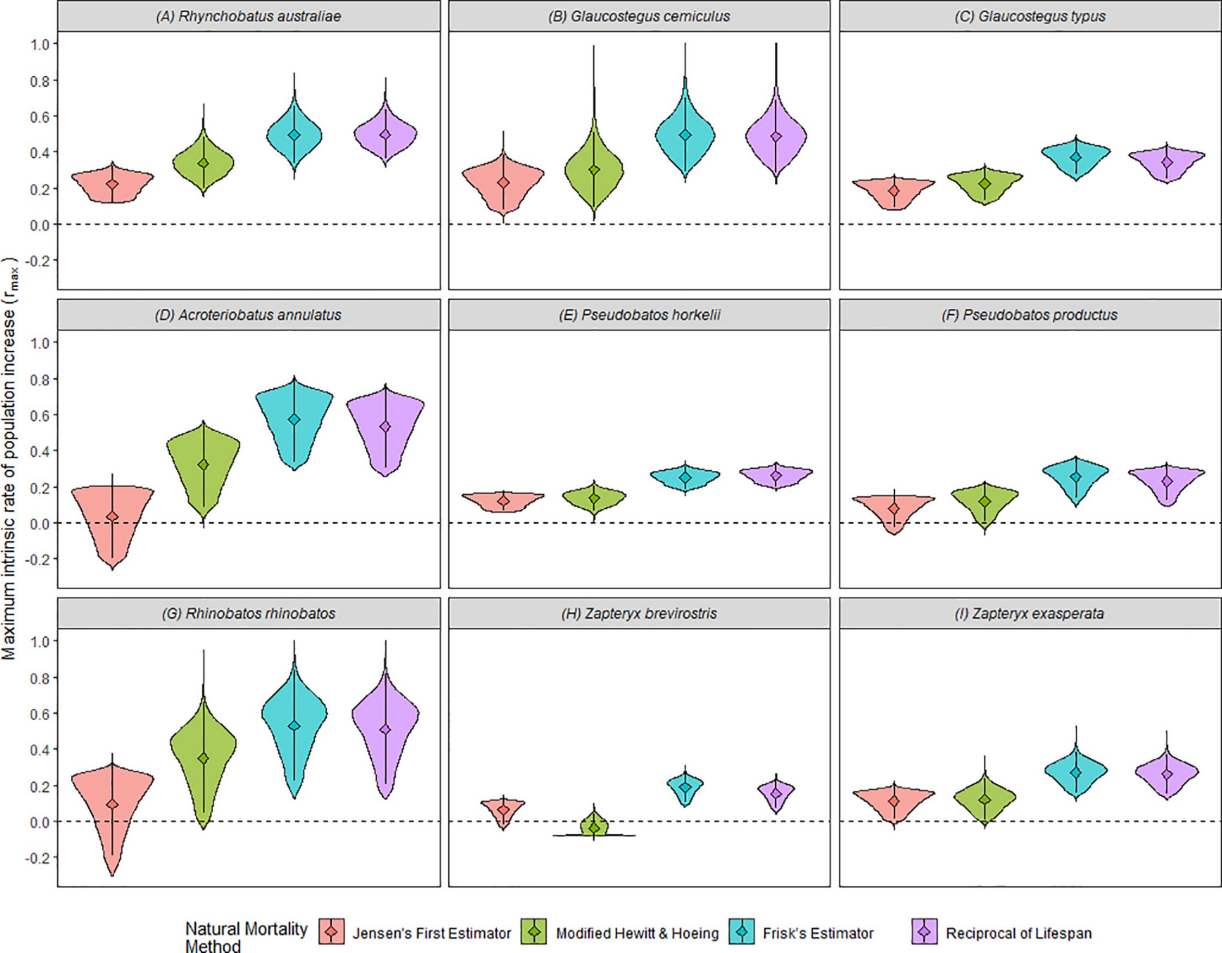
Values of *r*_*max*_ (year^-1^) for nine shovelnose ray species vary with different methods of estimating natural mortality. Which are Jensen’s First Estimator (red), modified Hoeing & Hewitt’s Estimator (yellow), Frisk’s Estimator (green), and Reciprocal of lifespan (blue). Means (triangle) and standard deviation (black line) are presented for each method. Species are (A) *R*. *australiae*, (B), *G*. *cemiculus*, (C) *G*. *typus*, (D) *A*. *annulatus*, (E) *P*. *horkelii*, (F) *P*. *productus*, (G) *R*. *rhinobatos*, (H) *Z*. *brevirostris*, and (I) *Z*. *exasperata*. Values below the black dashed line indicate implausible *r*_*max*_ estimates.

Estimates of maximum intrinsic rate of population increase for the nine species of shovelnose rays varied considerably among species, between families, and by the method of estimating natural mortality, ranging from 0.19 to 0.73 year^-1^ (25% - 95% quantiles) (Table 4). There was a high level of uncertainty in the annual reproductive output and age at maturity across all species (Fig 1). Uncertainty in the natural mortality values was low (Fig 1), but it resulted in high uncertainty in the *r_max_* estimates, which was highly influenced by the natural mortality estimator (Fig 2; Table 4).

**Table 4:** Estimates of *r*_*max*_ (year^-1^) for nine species of shovelnose rays using four methods of estimating natural mortality. The mean (± standard deviation S.D.) and 25% and 95% quantiles of *r*_*max*_ values are reported for each species and natural mortality estimator.

	Jensen’s First estimator				Hewitt & Hoeing’s estimator				Frisk's estimator				Reciprocal of lifespan estimator			
Species	25%	Mean	± S.D.	95%	25%	Mean	± S.D.	95%	25%	Mean	± S.D.	95%	25%	Mean	± S.D.	95%
*Rhynchobatus australiae*	0.18	0.22	0.050	0.30	0.29	0.34	0.069	0.46	0.44	0.50	0.077	0.63	0.45	0.49	0.067	0.61
*Glaucostegus cemiculus*	0.17	0.23	0.074	0.34	0.23	0.30	0.103	0.48	0.42	0.49	0.103	0.67	0.42	0.49	0.100	0.66
*Glaucostegus typus*	0.15	0.18	0.046	0.24	0.19	0.22	0.047	0.28	0.34	0.37	0.048	0.44	0.31	0.34	0.047	0.41
*Acroteriobatus annulatus*	-0.05	0.03	0.116	0.19	0.23	0.28	0.119	0.49	0.48	0.57	0.117	0.73	0.45	0.52	0.117	0.69
*Pseudobatos horkelii*	0.09	0.12	0.029	0.16	-0.11	0.13	0.035	0.25	0.23	0.25	0.032	0.29	0.24	0.26	0.031	0.31
*Pseudobatos productus*	0.04	0.08	0.053	0.14	0.08	0.12	0.055	0.19	0.22	0.25	0.056	0.33	0.19	0.23	0.053	0.30
*Rhinobatos rhinobatos*	0.00	0.10	0.143	0.27	0.25	0.35	0.153	0.57	0.43	0.53	0.154	0.75	0.41	0.51	0.152	0.73
*Zapteryx brevirostris*	0.04	0.06	0.040	0.11	-0.08	-0.04	0.038	0.03	0.16	0.19	0.042	0.25	0.13	0.16	0.041	0.21
*Zapteryx exasperata*	0.07	0.11	0.049	0.17	0.08	0.12	0.057	0.21	0.23	0.27	0.057	0.36	0.22	0.26	0.056	0.34

The ranges of *r_max_* for each species were relatively large as a result of the high uncertainty in the life-history parameters and method of estimating natural mortality (Fig 2). *Acroteriobatus annulatus* and *R*. *rhinobatos* had the largest range of *r_max_*, regardless of the natural mortality estimation method used (Fig 2; Table 4). *Pseudobatos horkelii* and *P*. *productus* had the smallest range of *r_max_* (Fig 2; Table 4). Frisk’s estimator, Maximum Age and Lifespan methods produced similar *r_max_* estimates for each species, with 7% or less difference between mean values (Fig 2; Table 4). The lowest *r_max_* values from every species were generated using the Jensen’s First estimator and modified Hewitt and Hoeing’s methods. These methods estimated negative *r_max_* values for *A*. *annulatus*, *P*. *horkelii*, and *Z*. *brevirostris* (Table 4; Fig 2). *Zapteryx brevirostris*, the smallest species in the study, had one of the lowest estimates of *r_max_*, across of natural mortality methods (Table 4).

**Fig 3:**
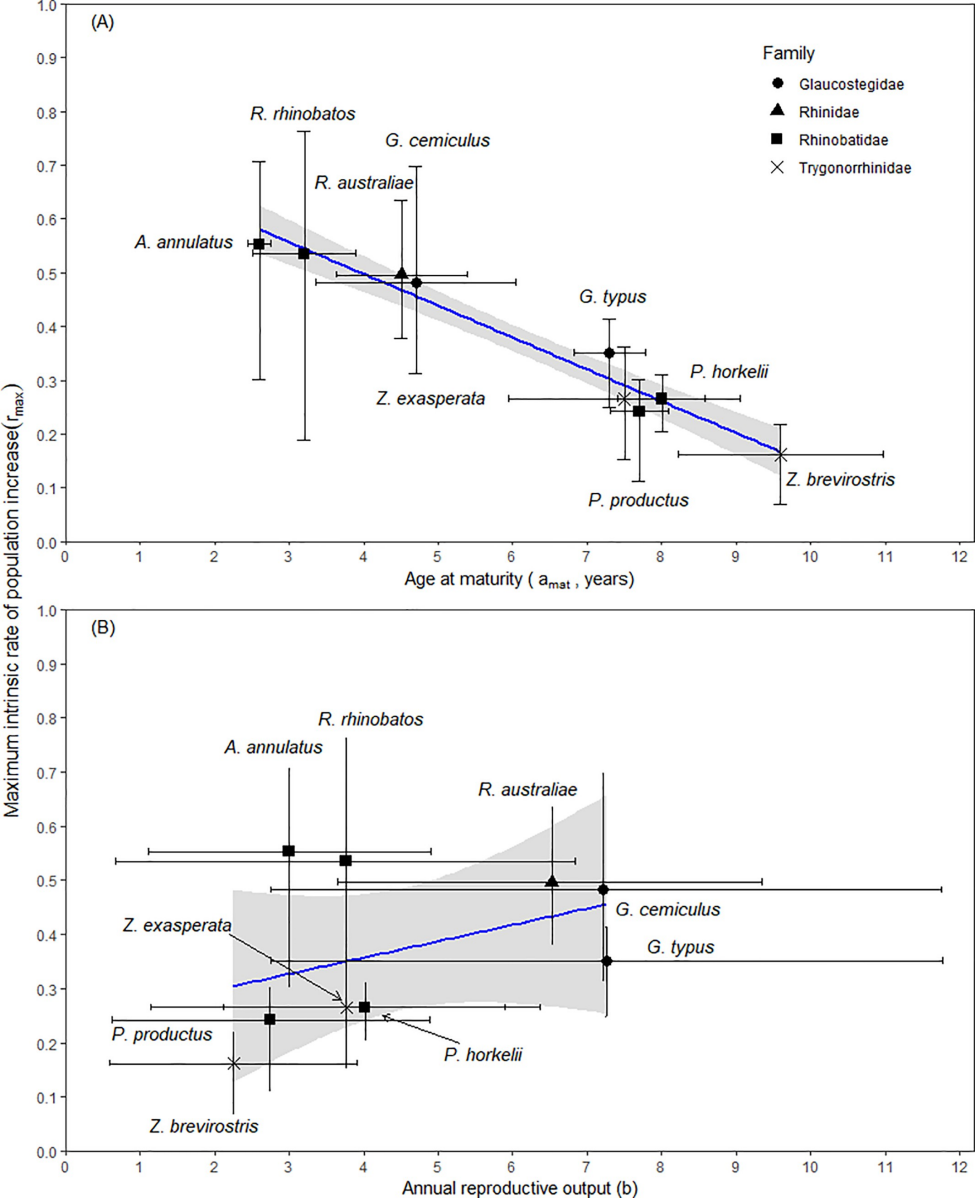
**Predicted value of *r*_*max*_ for the nine species of shovelnose rays in relation to their (A) age at maturity (*a*_*mat*_, years) and (B) annual reproduction rate of females (*b*).** The black lines encompass 95% of the values (2.5 and 97.5% quantiles). The reciprocal of lifespan natural mortality estimator to estimate *r*_*max*_. The shapes represent the four families; black circles represents the giant guitarfishes, Family Glaucostegidae; black triangles signifies the wedgefishes, Family Rhinidae; black squares represents guitarfishes, Family Rhinobatidae; and black crosses are banjo rays, Family Trygonorrhinidae.

**Fig 4:**
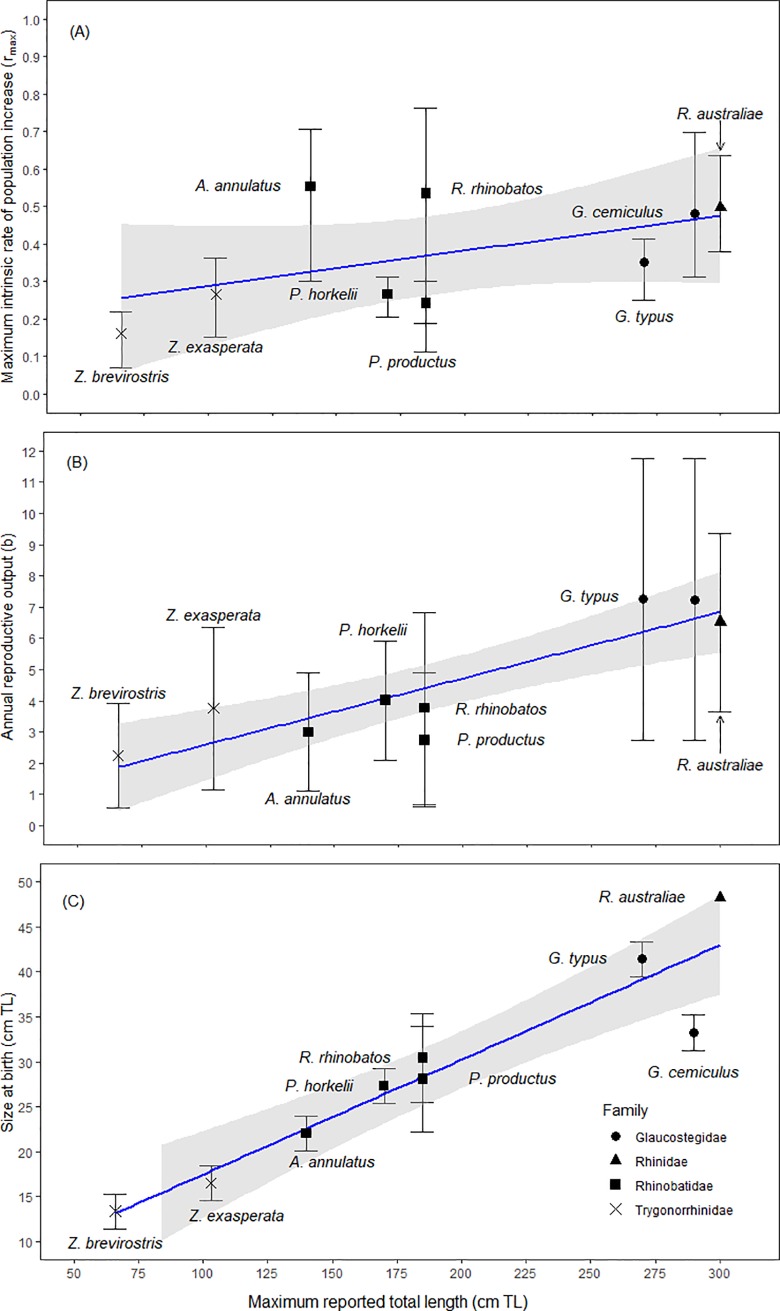
**Maximum size(cm TL) for the nine species of shovelnose rays in relation to the (A) median maximum intrinsic rate of population increase (*r*_*max*,_ year^-1^) using the reciprocal of lifespan to estimate natural mortality, (B) annual reproduction rate of females (*b*), and (C) size at birth (cm TL).** The black lines encompass 95% of the values (2.5 and 97.5% quantiles). The shapes represent the four families; black circles represents the giant guitarfishes, Family Glaucostegidae; black triangles signifies the wedgefishes, Family Rhinidae; black squares represents guitarfishes, Family Rhinobatidae; and black crosses are banjo rays, Family Trygonorrhinidae.

As the age at maturity decreased, the estimates of *r_max_* increased for the nine species of shovelnose rays (Fig 3A). The species with the highest median estimates of *r_max_*, *R*. *australiae*, *G*. *cemiculus*, *R*. *rhinobatos* and *A*. *annulatus* had the youngest age at maturity, while *Z*. *brevirostris* had the oldest age at maturity and lowest median estimate for *r_max_* (Fig 3A). The estimates of *r_max_* increased as the number of female offspring produced annually increased (Fig 3B). *Rhynchobatus australiae* and *G*. *cemiculus* had the highest annual reproductive output and *r_max_*, while *G*. *typus* had lower *r_max_* estimates but the same annual reproductive output as the two species (Fig 3B). *Rhinobatos rhinobatos*, *P*. *horkelii* and *Z*. *exasperata* had similar estimates of annual reproduction, yet *R*. *rhinobatos* had a higher estimate of *r_max_* than *P*. *horkelii* and *Z*. *exasperata* (Fig 3B). *Zapteryx brevirostris* had the lowest annual reproductive output and *r_max_* estimate (Fig 3B). Maximum rate of population growth increased with maximum size of the species (Fig 4A). The largest species (i.e. *R*. *australiae*, *G*. *cemiculus* and *G*. *typus*) were estimated to have a higher maximum rate of population increase than the smaller species in the order, such as *P*. *horkelii* and *Z*. *brevirostris* (Table 4; Fig 4A). The high maximum rate of population increase for the larger species was the result of the high mean annual reproductive outputs, large size at birth and an early age at maturity (Fig 4B and 4C). The smallest species, *Z*. *exasperata* and *Z*. *brevirostris*, had the lowest annual reproductive output and size at birth in relation to their maximum size (Fig 4B and 4C).

### Comparison of shovelnose ray *r_max_* estimates to other chondrichthyans

**Fig 5:**
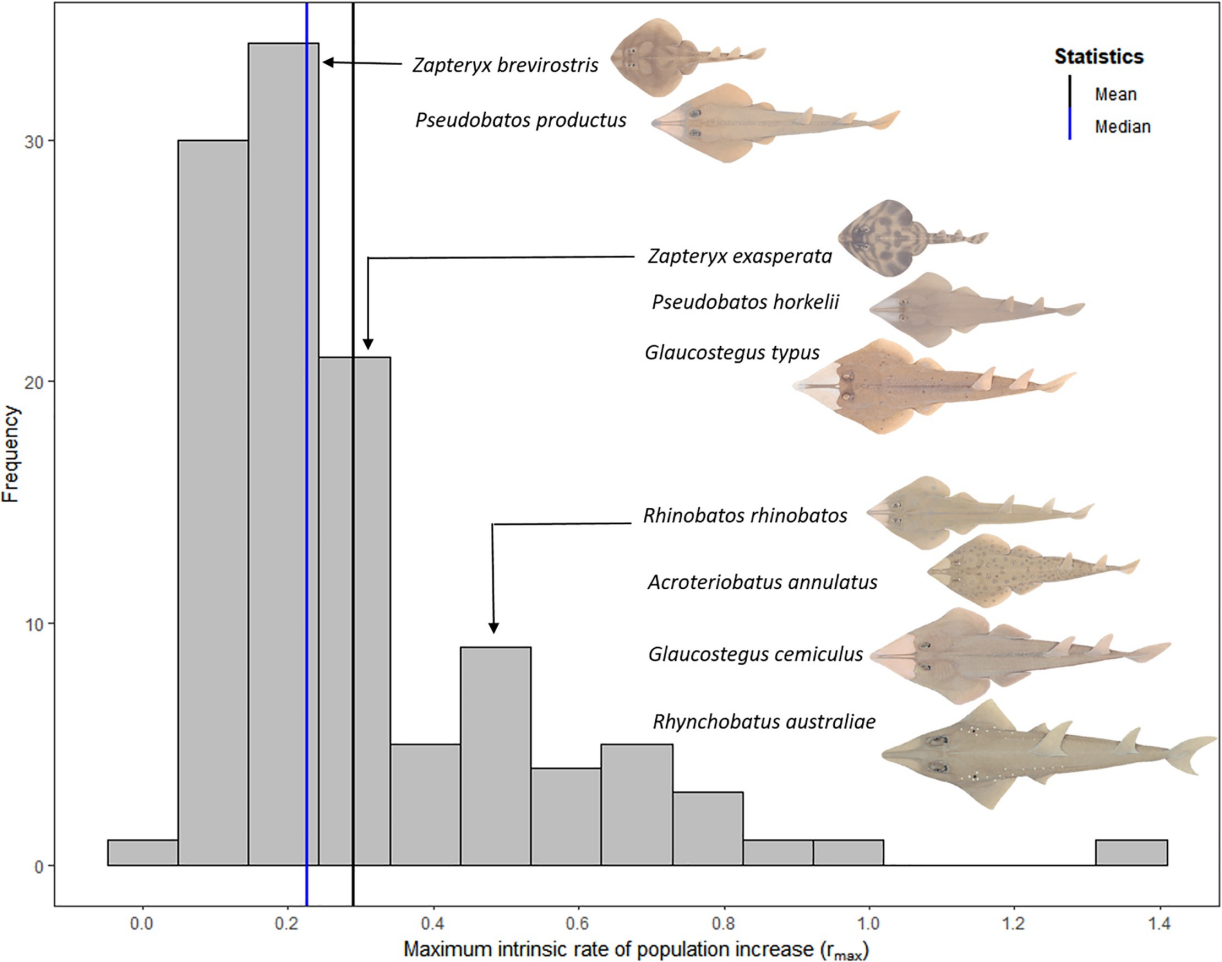
The frequency of the *r*_*max*_ values predicted for 115 chondrichthyans, including the nine shovelnose ray species. The reciprocal of lifespan natural mortality estimator was used to estimate *r*_*max*_ and species are grouped by their *r*_*max*_ values. Black line denote the mean (*r*_*max*_ = 0.30) and blue line represents the median (*r*_*max*_ = 0.23). The nine shovelnose rays species are displayed on the figure and species illustrations are from Last *et al*. [75].

The maximum intrinsic rate of population increase of the chondrichthyans ranged from 0.04 to 1.39 year^-1^, with the average *r_max_* estimate of 0.30 (Fig 5). Compared to the other chondrichthyans species, *Z*. *brevirostris* and *P*. *productus* have a below average *r_max_* estimates, while *Z*. *exasperata*, *P*. *horkelii*, and *G*. *typus* have medium *r_max_* estimates, and *R*. *rhinobatos*, *A*. *annulatus*, *G*. *cemiculus*, and *R*. *australiae* have a higher than average *r_max_* estimates (Fig 5, Table 4).

**Fig 6:**
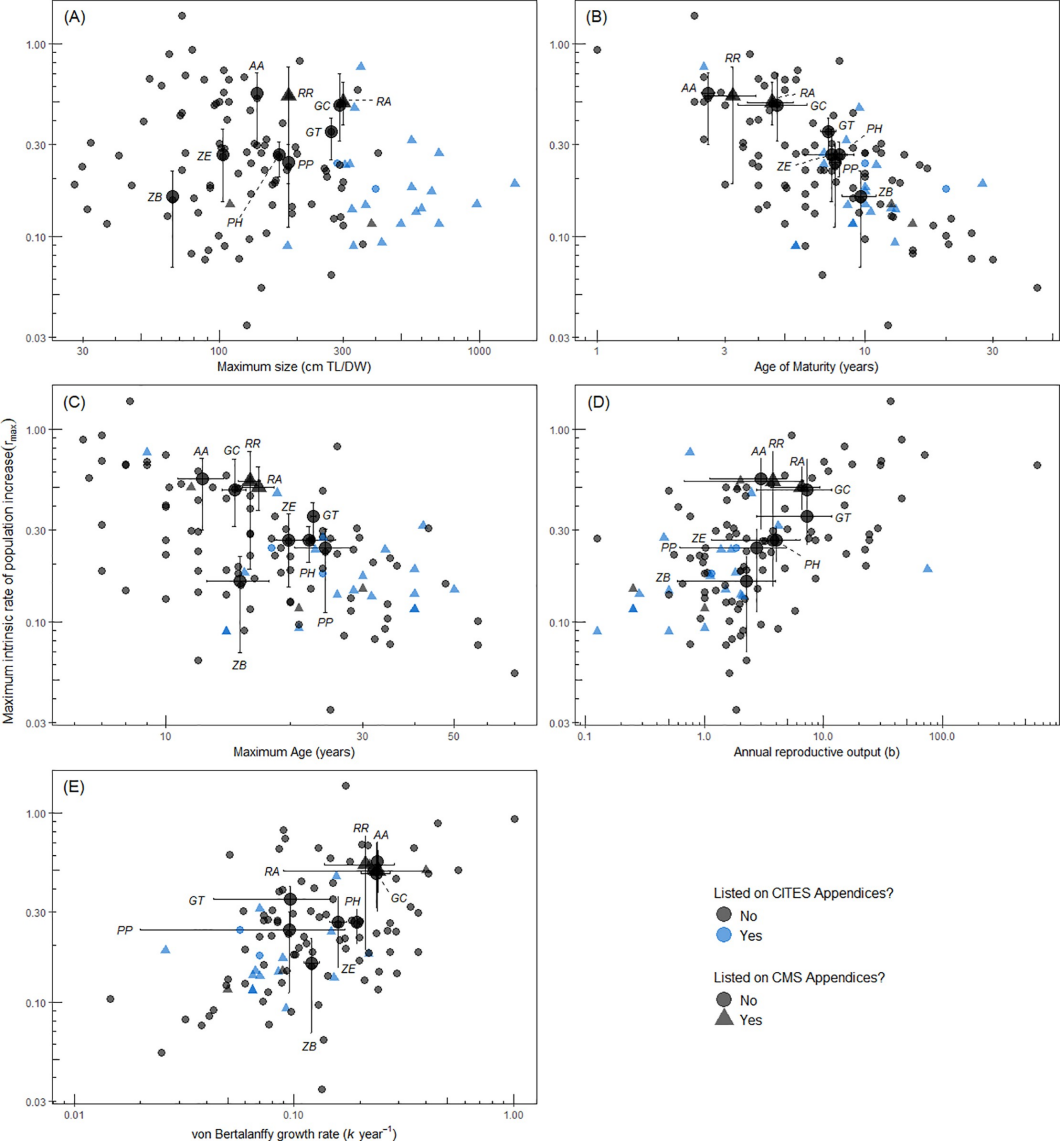
Estimates of *r*_*max*_ for 115 chondrichthyans, including the nine shovelnose rays species, compared with life history parameters. (A) maximum size (cm TL/DW), (B), age at maturity (*α*_*mat*_ years), (C) maximum age (*α*_*max*_, years), (D) annual reproductive output *b*, (E) the von Bertanlaffy growth coefficient (*k*, year^-1^). The nine shovelnose ray species labelled are: RA, *R*. *australiae;* GC, *G*. *cemiculus*; GT, *G*. *typus*; AA, *A*. *annulatus;* PH, *P*. *horkelii*; PP, *P*. *productus;* RR, *R*. *rhinobatos*, ZB, *Z*. *brevirostris;* ZE, *Z*. *exasperata*. The black lines encompass 95% of the values (2.5 and 97.5% quantiles). The median *r*_*max*_ value is reported, using the reciprocal of the lifespan method to estimate natural mortality. All axes are on a logarithmic scale. Species that are listed on CITES Appendix I or II are represented in blue, species listed on CMS Appendix I or II are represented as triangles. Species that are listed on neither CITES or CMS are indicated as grey circles.

*Rhynchobatus australiae*, *G*. *cemiculus* and *G*. *typus* had relatively high *r_max_* estimates, compared to species with similar maximum sizes (Fig 6A). *Pseudobatos horkelii*, *P*. *productus* and *Z*. *exasperata* had mid-range estimates of *r_max_* compared to species of a similar maximum size (Fig 6A). *Acroteriobatus annulatus* and *R*. *rhinobatos* had relatively high *r_max_*, while *Z*. *brevirostris* had a lower *r_max_* when compared to similar maximum sized species (Fig 6A). The majority of the largest chondrichthyan species for which *r_max_* are available are all listed on CITES and CMS, however they are not the least productive species (Fig 6A). *Acroteriobatus annulatus*, *G*. *cemiculus* and *R*. *australiae* mature at the youngest ages and had higher estimates of *r_max_*, compared to the other Rhinopristiformes and chondrichthyans (Fig 6B). *Acroteriobatus annulatus*, *R*. *rhinobatos*, *G*. *cemiculus* and *R*. *australiae* are among the chondrichthyans species with the lowest maximum age estimates, and hence high *r_max_* (Fig 6C). *Glaucostegus typus*, *Z*. *exasperata*, *P*. *horkelii* and *P*. *productus* have mid-range maximum ages compared to other species, while *Z*. *brevirostris* had a lower *r_max_* estimate compared to other species with a similar maximum age (Fig 6C). *Acroteriobatus annulatus*, *R*. *rhinobatos*, *G*. *cemiculus* and *R*. *australiae* have relatively higher *r_max_* estimates compared to species with similar annual reproductive output. *Zapteryx exasperata*, *P*. *horkelii* and *P*. *productus* are estimated to have a mid-range annual reproductive estimate, compared to species with similar *r_max_* (Fig 6D). *Glaucostegus typus* has a relatively high *r_max_* estimate compared to species with similar annual reproductive output, while *Z*. *brevirostris* has a low *r_max_* estimate compared to species with similar annual reproductive output (Fig 6D). *Acroteriobatus annulatus*, *R*. *rhinobatos*, *G*. *cemiculus* and *R*. *australiae* have fast somatic growth and a high *r_max_* in comparison to the other chondrichthyan species (Fig 6E). *Glaucostegus typus*, *Z*. *exasperata* and *P*. *horkelii* have a mid-range *r_max_* compared to species with similar growth rates, while *P*. *productus* and *Z*. *brevirostris* have a lower *r_max_* compared to other species with similar growth rates (Fig 6E).

## Discussion

Typically large-bodied marine animals are associated with factors of vulnerability, such as lower intrinsic rate of population growth, late maturity, and dependence on vulnerable habitat, while smaller-bodied species are linked to factors providing resilience, including faster population growth and early maturity [1, 72, 112]. The productivity of shovelnose rays was similar to four sawfish species, which despite their large size (ranging from 318 – 700 cm TL) have been estimated to have a relatively high productivity for elasmobranchs [51]. The positive relationship between maximum size and maximum intrinsic rate of population growth for seven out of nine shovelnose ray species in this study is unusual among elasmobranchs [113]. This relationship is being driven by the positive relationship between body size and litter size, as the litter size increases with the maximum size of these rays. These findings for these species contrasts other multi-species comparative studies, such as Dulvy *et al*. [13], where the maximum intrinsic rate tends to decrease with increasing maximum size. *Acroteriobatus annulatus* and *R*. *rhinobatos* did not fall within this positive relationship due to their young age at maturity, fast somatic growth, and high annual reproductive output [75]. While body size has been used to predict extinction risk in elasmobranchs, with the larger species predicted to be most at risk of extinction [1], this may not be the case for some shovelnose rays. Additionally, other studies have found little [66, 72] to no correlation [5] between body size and rate of population increase. The relationship between body size and rate of population growth has been hypothesised to be the result of correlations between body size and other more influential life-history traits such as age at maturity and litter size [114, 115].

The estimates of *r_max_* are sensitive to increasing variation in age at maturity [71]. The early maturity of shovelnose rays, particularly compared to other species of similar size, as well as the increasing litter size with increasing body size, help to explain the relatively high *r_max_* estimates for this group. The larger body size of wedgefishes and giant guitarfishes allows these species to produce numerous and large offspring in relation to their maximum size. In contrast, the guitarfishes and banjo rays have smaller birth size and smaller litters relative to their maximum size. Larger offspring will likely have a greater survival probability than the smaller offspring of species with a similar *r_max_* [71]. For long-lived species, juvenile survival is a key contributor to the population growth rate [66]. While the model used in this study incorporates juvenile survival, it also assumes that juvenile mortality is equal to adult mortality [73]. Juveniles, as well as neonates (age 0) tend to have higher mortality rates than adults [116], which then can vary with local differences in habitat [117]. This assumption of equal mortality is likely to result in conservative estimates of *M* [73]. The differential neonate and juvenile mortality among species was not accounted for in this model, but should be the focus of further study [71].

Natural mortality, referring to the death of individuals in the population from natural causes such as predation, disease and old age [106], is one of the most important parameters in fisheries and conservation modelling, yet it is one of the hardest to estimate [67, 118, 119]. While in some models uncertainty in the natural mortality parameter has little influence on *r_max_* [71], different estimators can have substantial effects on *r_max_* values [119]. Frisk’s estimator and Reciprocal of life span are more suited for elasmobranchs, given they have a relatively high juvenile survival [66, 73]. Taking into account juvenile mortality, *r_max_* estimates produced by these two natural mortalities suggest these estimators are more plausible and may be the more appropriate methods for elasmobranchs. In contrast the Jensen’s First Estimator [109] and the modified Hewitt and Hoeing method [110] were explicitly designed for adult mortality and systematically resulted in negative value of *r_max_* for five out of the nine species of shark-like ray species. The biologically implausible estimates were also demonstrated in Pardo *et al*. [73], and are likely the consequence of overestimating natural mortality (e.g. > 0.1 year^-1^) for these species, particularly when the annual reproductive output is low (e.g. *b* < 5) and age at maturity is high [71, 73]. It is therefore likely that Jensen’s First Estimator and the modified Hewitt and Hoeing are less appropriate methods of estimating natural mortality for chondrichthyans. There is considerable debate as to which empirical model should be used to estimate adult natural mortality, as there are numerous and diverse approaches using life-history information to estimate this parameter [118, 120]. However, identifying, or improving the best indirect estimator would require data-intensive methods, such as catch data to analyse catch curves, mark re-capture experiments, virtual population analysis, or fully integrated stock assessments [120]. These methods all require extensive prior knowledge of the biology of the species that is lacking for many chondrichthyan species. Presenting the results from multiple natural mortality estimators provides a better understanding of the uncertainty associated with the maximum intrinsic rate of population increase.

The greatest obstacle to accurately estimate *r_max_* and natural mortality is the accuracy of the biological information used [103]. The use of inaccurate surrogate information can reduce the accuracy of the demographic models [103, 121, 122]. Of the 56 species across the four families of shovelnose rays, only nine species had sufficient information to estimate their maximum intrinsic rate of population increase, and with relatively high levels of uncertainty associated with the life-history parameters and small sample sizes. For example, there were only two age and growth studies for wedgefishes and giant guitarfishes, one from the eastern coast of Australia for *R*. *australiae* and *G*. *typus* [74], and one from Central Mediterranean Sea for *G*. *cemiculus* [82]. Neither study estimated age at maturity, nor aged individuals at the maximum sizes. Given that the age at maturity is a pivotal parameter when estimating *r_max_*, yet highly uncertain for all shovelnose rays examined, these estimates must be taken with caution. Furthermore, numerous reviews have reported sampling biases and failures in ageing protocols, including lack of validation [123, 124] that often result in overestimation or underestimate of age and growth parameters [125]. As there has been no validation studies in the ages of wedgefishes, guitarfishes, and banjo rays, the maximum ages for these species are likely to be underestimated, while the age at maturity estimates could also be inaccurate. This can lead to inaccurate estimates of natural mortality and *r_max_* [103, 126]. The information on the reproductive biology for Rhinopristiformes is limited, but is more available for species in the guitarfishes Rhinobatidae and Trygonorrhinidae families. For example, there is evidence that species such as *P*. *productus*, *P*. *horkelii*, and Z. *exasperata* employ embryonic diapause or delayed development [99, 127], potentially as a result of unfavourable environmental conditions [128] or sex segregation [129]. Simpfendorfer [130] hypothesised that diapause allowed another elasmobranch species (*Rhizoprionodon taylori*) to have larger litter sizes than other similar sized species in the same family (Carcharhinidae). Capture-induced parturition (premature birth or abortion) during sampling is possible for elasmobranchs and can result in the underestimation of litter sizes [131]. As possibility of diapause and capture induced parturition was not able to be taken into account during this study, the breeding interval and annual reproductive output may be inaccurate, and it could result in an inappropriate maximum intrinsic rate of population growth. Directing research efforts to obtain data from more species, as well as improving the accuracy of life-history parameters for data-poor species, such as age at maturity and annual reproductive output, would be the most pragmatic option to improve the accuracy of *r_max_* for shovelnose rays.

Measuring the population productivity of a species allows for a greater understanding of the species’ ability to recover from declines and provides the demographic basis for evaluating the sustainability of fisheries and trade [103, 132]. The unregulated fishing pressure that most shovelnose ray species currently experience is likely unsustainable [19, 36]. Yet, there are minimal regional and national level management by countries within the ranges of shovelnose rays. To reduce fishing mortality, conserve populations and allow for recovery, a suite of management measures will be required including species protection, spatial management, bycatch mitigation, and harvest strategies [24].

International trade of highly-valued fins is considered a major driver of over-exploitation for shovelnose rays [24, 57] and the use of trade controls through CITES listings may be an effective way to encouraging better management of shovelnose ray species. In 2019, the wedgefishes (Rhinidae) and giant guitarfishes (Glaucostegidae) were listed on the CITES Appendix II [133]. Any Parties that wishes to export products from these rays, requires a NDF, which provides evidence that the populations that supply the trade are sustainable. In addition, CITES, unlike many other international agreements, has the capacity to enforce its actions through a Review of Significant Trade and possible trade suspensions, in conjunction with national-level enforcement and compliance measures [55]. The recent CITES Appendix II listing provides an opportunity to gather information through the CITES database, which holds all permitted exports, re-exports and imports of Appendix II species. As other commercially important elasmobranch species are listed on CITES, a number of capacity building tools are available for Parties for the implementation and enforcement of elasmobranchs on Appendices, including an elasmobranch specific information portal [134], and a new species identification guide for wedgefishes and giant guitarfishes [135]. International agreements such as CITES and CMS are only one step needed to reduce threats of these species in international trade, recover populations, ensure sustainable resource use, and are designed to be complementary to existing national and regional management [55]. Fisheries are complex social-ecological systems, and successful management will require significant improvements in governance across local, global and regional scales [57]. After the enactment of national and international management measures to reduce fishing mortality, the theoretical maximum intrinsic rate of population increase of some species of shovelnose rays (i.e. *R*. *australiae*, *G*. *cemiculus*, *G*. *typus*), infers that they have the biological capacity to recover relatively quickly from the reported population declines.

## Conclusion

Using current life-history data, incorporating uncertainty in parameters, and taking into account juvenile mortality, this study provides the first analysis into the population productivity for nine species from four families of Rhinopristiformes. Compared to other chondrichthyans, the larger wedgefish and giant guitarfishes were found to be potentially productive species, while the smaller guitarfishes and banjo rays were less productive. The maximum intrinsic rate of population increase varied with the different natural mortality estimator, yet it also appears to increase with increasing maximum size for the four families, which is counter to most studies of shark populations. There was considerable uncertainty in the age at maturity and annual reproductive output for all species. There is a need for better life-history information for these data-poor species, as there was only nine of out 56 species with sufficient life-history information. We recommend presenting the results from multiple natural mortality estimators to provide a greater understanding of the uncertainty for the maximum intrinsic rate of population increase. It appears that wedgefishes and giant guitarfishes could, theoretically, recover from population depletion faster than guitarfishes and banjo rays, if fishing mortality is kept low. Extensive regional, national and international fisheries management strategies, including the regulation of international trade through CITES, will be required to address the overfishing of these species, and may help to achieve positive conservation outcomes. The results of this study provides guidance to help implement management and conservation measures, while highlighting the lack of information available for these species.

## Supporting information

S1 Appendix Re-estimating the three parameter von Bertalanffy growth rate of *Rhynchobatus australiae* and *Glaucostegus typus* from White *et al*. [74]. (DOCX)

S2 Appendix Predicted values of maximum intrinsic rate of population increase (*r*_*max*_) for nine shovelnose ray species when including uncertainty the other three natural mortality methods. (DOCX)

S1 Table Maximum intrinsic rate of population increase (*r*_*max*_) estimates, life-history values and sources used to estimate *r*_*max*_ for additional chondrichthyan species added to the comparison analysis. The natural mortality method used was the reciprocal of the lifespan method. The values included are the maximum size (*L*_*max*_ in centimetres total length/disk width, cm TL/DW), von Bertalanffy growth coefficient (*k*, year^-1^), age at maturity (*α*_*mat*_, years), reported maximum age (*α*_*max*_, years), litter size (*l*), breeding interval (*i*, years), annual reproductive output of females (*b*). Included is whether the species are listed on the appendixes of Convention of International Trade of Endangered Species (CITES, yes or no) and/or Convention on the Conservation of Migratory Species of Wild Animals (CMS, yes or no). The ‘na’ indicates parameter was not available from literature.
(DOCX)
