# Supplementary material for: Population productivity of shovelnose rays: Inferring the potential for recovery
Source: PLoS One. 2019 Nov 21;14(11):e0225183. doi: 10.1371/journal.pone.0225183 (PMC6872150; doi:10.1371/journal.pone.0225183)
Supplement: S1 Appendix — (DOCX) [file pone.0225183.s001.docx]

**D’Alberto et al (2019) Population productivity of shovelnose rays: inferring the potential for recovery**

**S1 Appendix.** Re-estimating the three parameter von Bertalanffy growth rate of *Rhynchobatus australiae* and *Glaucostegus typus* from White *et al.* [1]

*Method*

Published observed and back calculated length at age data was extracted from the [1] Figure 4 for combined sexes of *Rhynchobatus australiae*, and combined sexes of *Glaucostegus typus* using the program, Data Thief [2]. Males and females samples were combined in the study and therefore, the estimates for combined sexes are presented here. The extracted length at age for the observed and back calculated data was fitted to the three parameter von Bertalanffy growth model (VBGF, [3]) in the R statistical environment [4] as:

$$L_{t}=L_{0}+(L_{\infty}-L_{0})(1-\exp(-kt))$$

where *L_t_* is length at age *t*, *L_0_* is length at age 0, *L_∞_* is asymptotic length, and *k*  is the von Bertalanffy growth rate. The model was fitted using the biologically relevant length at birth parameter (*L_0_*), instead of the time at size zero parameter (*t_0_*). Parameter estimates were estimated using non-linear least-square regression methods. The standard errors for the parameter estimates were calculated using bootstrapping method with the ‘nlstools’ package in R.

*Results*

The extracted ages for *R. australiae* and *G. typus* ranged from 1 – 11 years and 0 – 18 years, respectively. Length estimates ranged from 51 – 235 cm TL and 51 – 286 cm TL for *R. australiae* and *G. typus,* respectively. The data points extracted from the Figure 4 for the *G. typus* observed data was greater than reported in text of 23 samples, while there was only 45 data points extracted for the observed *R. australiae* data (S1 Table). Given the considerable uncertainty associated with this data, the lowest *k* value was used as the minimum *k* estimated in the model for *R. australiae* and G*. typus* (Table 1).

**Table 1.** Summary of the three parameter von Bertalanffy estimates for the observed length-at-age data and back calculated data for *R. australiae* and *G. typus*, from Eastern Australia. Length-at-age was extracted from White *et al.* [1], using the program Data Thief [2]. *n* refers to the sample size, *L_∞_* the asymptotic length (± standard error S.E. in centimetres total length, cm TL), *k* is the von Bertalanffy growth parameter (S.E. ± year^-1^) and *L_0_* is the size at birth (± S.E. cm TL).

|  | *n* | *L_∞_* (± S.E. cm TL) | *k* (± S.E. year^-1^) | *L_0_* (± S.E. cm TL) |
| --- | --- | --- | --- | --- |
| **Observed data** | | | | |
| *Rhynchobatus australiae* | 45 | 317.8 ± 213.6 | 0.082 ± 0.101 | 52.47 ± 21.82 |
| *Glaucostegus typus* | 60 | 402.1 ± 123.9 | 0.059 ± 0.031 | 49.85 ± 11.34 |
|  |  |  |  |  |
| **Back calculated data** | | | | |
| *Rhynchobatus australiae* | 111 | 232.2 ± 40.29 | 0.133 ± 0.521 | 44.45 ± 9.684 |
| *Glaucostegus typus* | 101 | 500.8 ± 108.5 | 0.040 ± 0.129 | 39.16 ± 5.912 |

**Literature cited**

1. White J, Simpfendorfer CA, Tobin AJ, Heupel MR. Age and growth parameters of shark-like batoids. Journal of Fish Biology. 2014;84(5):1340-53. Epub 2014/04/08. doi: 10.1111/jfb.12359. PubMed PMID: 24702252.

2. Tummers B. DataThief III 2006 [cited 2019]. Available from: <https://datathief.org/>.

3. von Bertalanffy L. A quantitative theory of organic growth (inquiries on growth laws II). Human Biology. 1938;10:181 - 213.

4. Team RC. R: A Language and Environment for Statistical Computing. 2016.
