# Supplementary material for: Population productivity of shovelnose rays: Inferring the potential for recovery
Source: PLoS One. 2019 Nov 21;14(11):e0225183. doi: 10.1371/journal.pone.0225183 (PMC6872150; doi:10.1371/journal.pone.0225183)
Supplement: S2 Appendix — (DOCX) [file pone.0225183.s002.docx]

**D’Alberto et al (2019) Population productivity of shovelnose rays: inferring the potential for recovery**

**S2 Appendix.** Predicted values of maximum intrinsic rate of population increase (*r_max_*) for nine shovelnose ray species when including uncertainty the other three natural mortality methods.


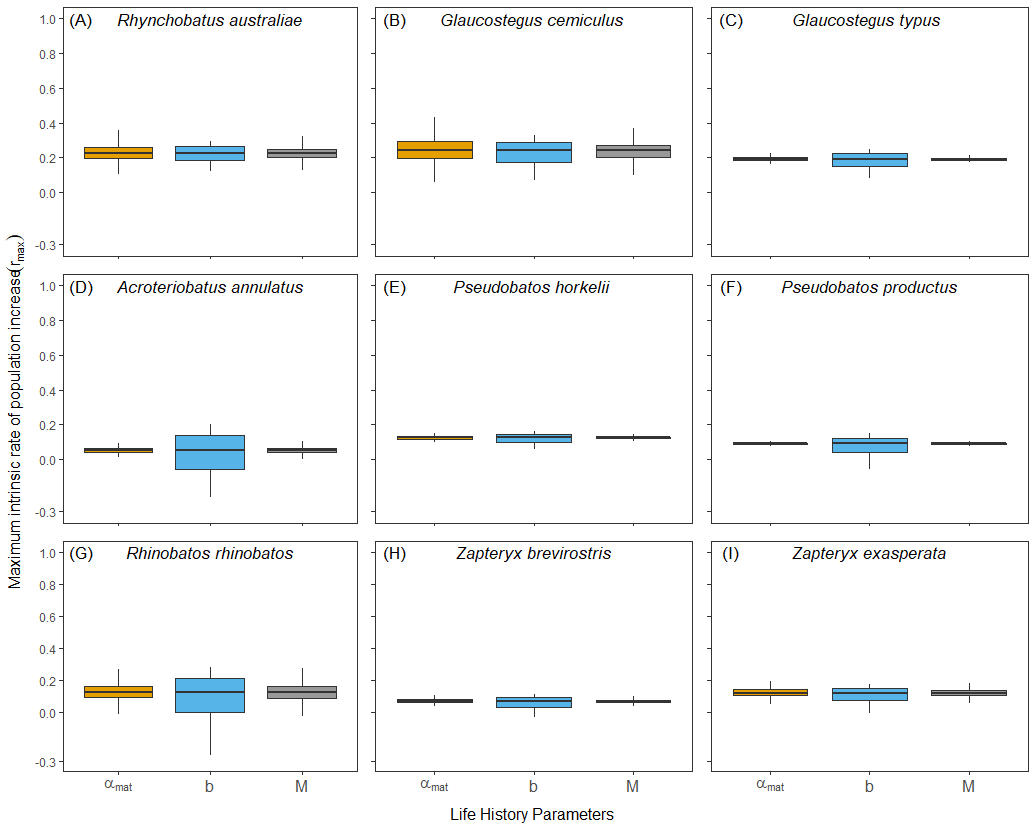


**Figure 1.** Predicted values of maximum intrinsic rate of population increase (*r_max_*) for nine shovelnose rays species when including uncertainty in age at maturity (*α_mat_*, first/orange boxplot), annual reproductive output (*b,* middle/blue boxplot), and Jensen’s natural mortality estimator (*M*, last/grey boxplot). Species are (A) *R. australiae,* (B), *G. cemiculus,* (C) *G. typus,* (D) *A. annulatus,* (E) *P. horkelii*, (F) *P. productus,* (G) *R. rhinobatos,* (H) *Z. brevirostris*, and (I) *Z. exasperata*. Boxes indicate median, 25 and 75% quantiles, whereas the lines encompass 95% of the values (2.5 and 97.5% quantiles).


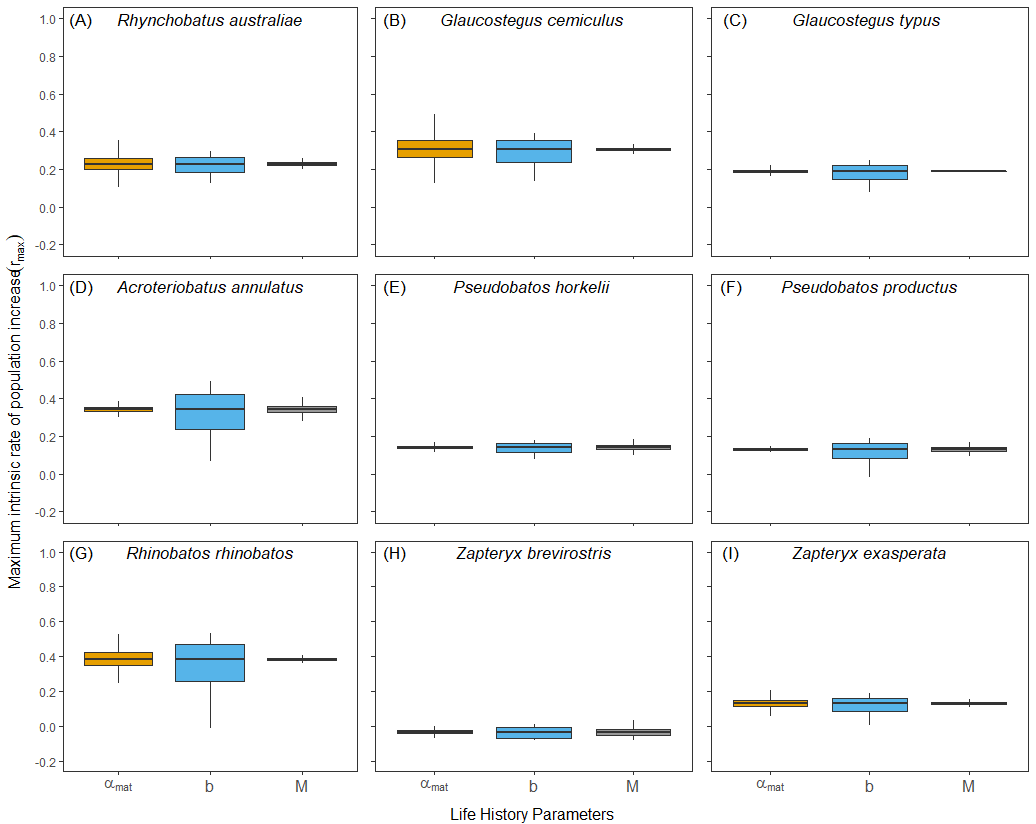


**Figure 2.** Predicted values of maximum intrinsic rate of population increase (*r_max_*) for nine shovelnose rays species when including uncertainty in age at maturity (*α_mat_*, first/orange boxplot), annual reproductive output (*b,* middle/blue boxplot), and modified Howitt & Hewitt’s natural mortality estimator (*M*, last/grey boxplot). Species are (A) *R. australiae,* (B), *G. cemiculus,* (C) *G. typus,* (D) *A. annulatus,* (E) *P. horkelii*, (F) *P. productus,* (G) *R. rhinobatos,* (H) *Z. brevirostris*, and (I) *Z. exasperata*. Boxes indicate median, 25 and 75% quantiles, whereas the lines encompass 95% of the values (2.5 and 97.5% quantiles).


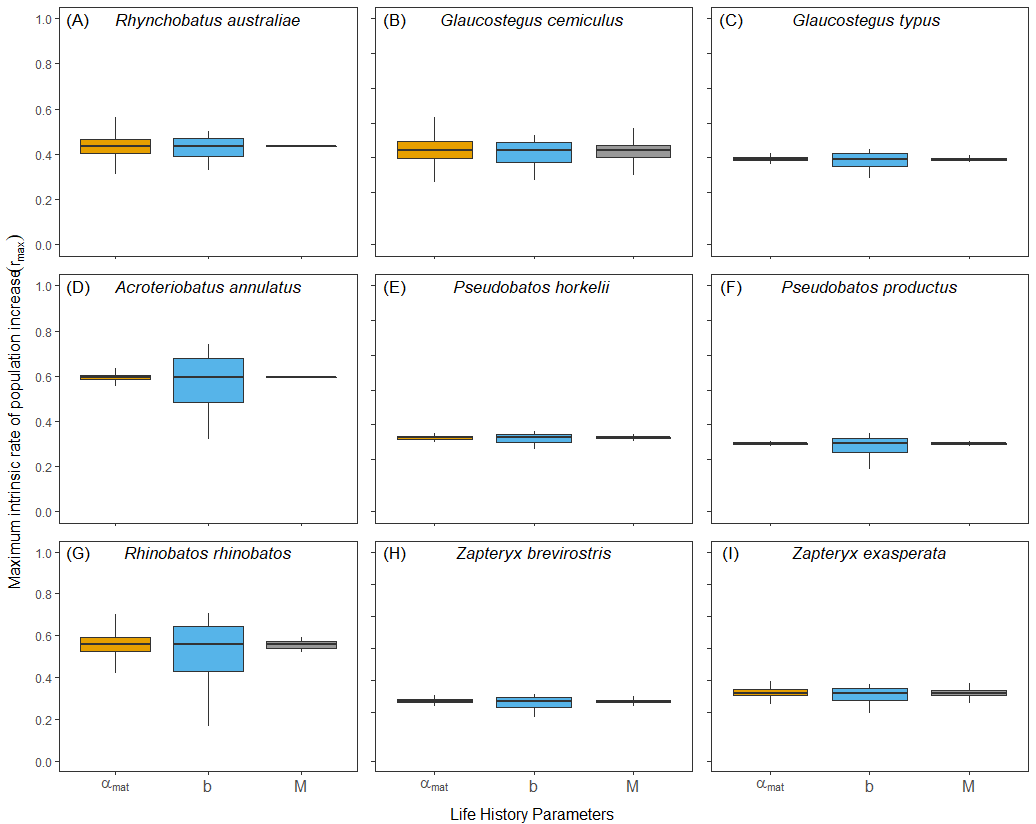


**Figure 3.** Predicted values of maximum intrinsic rate of population increase (*r_max_*) for nine shovelnose rays species when including uncertainty in age at maturity (*α_mat_*, first/orange boxplot), annual reproductive output (*b,* middle/blue boxplot), and Frisk’s natural mortality estimator (*M*, last/grey boxplot). Species are (A) *R. australiae,* (B), *G. cemiculus,* (C) *G. typus,* (D) *A. annulatus,* (E) *P. horkelii*, (F) *P. productus,* (G) *R. rhinobatos,* (H) *Z. brevirostris*, and (I) *Z. exasperata*. Boxes indicate median, 25 and 75% quantiles, whereas the lines encompass 95% of the values (2.5 and 97.5% quantiles).
