# Supplementary material for: Population productivity of shovelnose rays: Inferring the potential for recovery
Source: PLoS One. 2019 Nov 21;14(11):e0225183. doi: 10.1371/journal.pone.0225183 (PMC6872150; doi:10.1371/journal.pone.0225183)
Supplement: S1 Table — The natural mortality method used was the reciprocal of the lifespan method. The values included are the maximum size (Lmax in centimetres total length/disk width, cm TL/DW), von Bertalanffy growth coefficient (k, year-1), age at maturity (αmat, years), reported maximum age (αmax, years), litter size (l), breeding interval (i, years), annual reproductive output of females (b). Included is whether the species are listed on the appendixes of Convention of International Trade of Endangered Species (CITES, yes or no) and/or Convention on the Conservation of Migratory Species of Wild Animals (CMS, yes or no). The ‘na’ indicates parameter was not available from literature. (DOCX) [file pone.0225183.s003.docx]

**D’Alberto et al (2019) Population productivity of shovelnose rays: inferring the potential for recovery**

**S1 Table.** Maximum intrinsic rate of population increase (*r_max_*) estimates, life history values and sources used to estimate *r_max_* for additional chondrichthyan species added to the comparison analysis. The natural mortality method used was the reciprocal of the lifespan method. The values included are the maximum size (*L_max_* in centimetres total length/disk width, cm TL/DW), von Bertalanffy growth coefficient (*k*, year^-1^), age at maturity (*α_mat_*, years), reported maximum age (*α_max_*, years), litter size (*l*), breeding interval (*i*, years), annual reproductive output of females (*b*). Included is whether the species are listed on the appendixes of Convention of International Trade of Endangered Species (CITES, yes or no) and/or Convention on the Conservation of Migratory Species of Wild Animals (CMS, yes or no). The ‘na’ indicates parameter was not available from literature.

| **Order** | **Family** | **Species** | **CITES** | **CMS** | **L_max_ (cm TL/DW)** | ***k* (year^-1^)** | ***α_mat_* (years)** | ***α_max_* (years)** | **average lifespan** | ***l*** | ***i* (years)** | ***b*** | ***r_max_*** | **Source** |
| --- | --- | --- | --- | --- | --- | --- | --- | --- | --- | --- | --- | --- | --- | --- |
| Carchariniformes | Sphyrnidae | *Sphyrna mokarran* | Yes | Yes | 550.0 | 0.070 | 8.5 | 42.0 | 25.2 | 6.0 | 2 | 4.15 | 0.314 | [1, 2] |
| Carchariniformes | Sphyrnidae | *Sphyrna zygaena* | Yes | No | 400.0 | 0.070 | 20.0 | 24.0 | 22.0 | 17.3 | 1 | 1.15 | 0.177 | [3] |
| Lamniformes | Alopiidae | *Alopias vulpinus* | Yes | Yes | 573.0 | 0.153 | 10.5 | 31.5 | 21.0 | 1.1 | 2 | 2.08 | 0.134 | [4-6] |
| Myliobatiformes | Dasyatidae | *Maculabatis astra* | No | No | 80.0 | 0.073 | 9.0 | 47.5 | 28.2 | 1.0 | 1 | 0.65 | 0.158 | [7, 8] |
| Myliobatiformes | Dasyatidae | *Neotrygon picta* | No | No | 32.0 | 0.080 | 3.5 | 43.3 | 23.4 | 1.0 | 1 | 1.75 | 0.306 | [9, 10] |
| Myliobatiformes | Mobulidae | *Mobula alfredi* | Yes | Yes | 500.0 | 0.065 | 9.0 | 40.0 | 24.5 | 0.4 | 1.5 | 0.25 | 0.116 | [11] |
| Myliobatiformes | Mobulidae | *Mobula birostris* | Yes | Yes | 700.0 | 0.065 | 9.0 | 40.0 | 24.5 | 0.4 | 1.5 | 0.25 | 0.116 | [11] |
| Myliobatiformes | Mobulidae | *Mobula tarapacana* | Yes | Yes | 328.0 | na | 5.5 | 14.0 | 9.8 | 0.5 | 1 | 0.50 | 0.089 | [11, 12] |
| Myliobatiformes | Mobulidae | *Mobula thurstoni* | Yes | Yes | 183.0 | na | 5.5 | 14.0 | 9.8 | 0.5 | 1 | 0.13 | 0.089 | [13] |
| Rhinopristiformes | Glaucostegidae | *Glaucostegus cemiculus* | No | No | 290.0 | 0.237 | 4.7 | 14.7 | 9.7 | 7.0 | 1 | 1.88 | 0.486 | [14-18] |
| Rhinopristiformes | Glaucostegidae | *Glaucostegus typus* | No | No | 270.0 | 0.150 | 7.3 | 18.4 | 12.9 | 7.0 | 1 | 0.75 | 0.333 | [14, 19, 20] |
| Rhinopristiformes | Pristidae | *Anoxypristis cuspidata* | Yes | Yes | 350.0 | na | 2.5 | 9.0 | na | 6.2 | 1 | 0.75 | 0.757 | [21] |
| Rhinopristiformes | Pristidae | *Pristis clavata* | Yes | Yes | 318.0 | na | 7.0 | 34.0 | na | 1.8 | 2 | 1.38 | 0.236 | [21] |
| Rhinopristiformes | Pristidae | *Pristis pectinata* | Yes | Yes | 550.0 | 0.219 | 10.0 | 15.5 | 12.7 | 3.0 | 2 | 1.83 | 0.180 | [21] |
| Rhinopristiformes | Pristidae | *Pristis zijsron* | Yes | Yes | 700.0 | na | 7.0 | 24.0 | na | 3.0 | 2 | 0.46 | 0.272 | [21] |
| Rhinopristiformes | Rhinidae | *Rhynchobatus australiae* | No | Yes | 300.0 | 0.400 | 4.5 | 11.5 | 8.0 | 7.0 | 1 | 1.88 | 0.468 | [14, 20] |
| Rhinopristiformes | Rhinobatidae | *Acroteriobatus annulatus* | No | No | 140.0 | 0.240 | 2.6 | 10.9 | 6.8 | 3.0 | 1 | 3.63 | 0.522 | [14, 22] |
| Rhinopristiformes | Rhinobatidae | *Pseudobatos horkelii* | No | No | 170.0 | 0.194 | 8.0 | 22.2 | 15.1 | 4.0 | 1 | 3.25 | 0.261 | [14, 23] |
| Rhinopristiformes | Rhinobatidae | *Pseudobatos productus* | No | No | 185.0 | 0.095 | 7.7 | 24.3 | 16.0 | 3.0 | 1 | 3.62 | 0.230 | [14, 24-26] |
| Rhinopristiformes | Rhinobatidae | *Rhinobatos rhinobatos* | No | Yes | 185.0 | 0.222 | 3.2 | 16.0 | 12.0 | 3.8 | 1 | 2.00 | 0.537 | [14, 27-32] |

**Literature cited**

1. Stevens JD, Lyle JM. Biology of three hammerhead sharks (*Eusphyra blochii, Sphyrna mokarran* and *S. lewini*) from northern Australia. Marine and Freshwater Research. 1989;40(2):129-46.

2. Harry AV, Macbeth WG, Gutteridge AN, Simpfendorfer CA. The life histories of endangered hammerhead sharks (Carcharhiniformes, Sphyrnidae) from the east coast of Australia. Journal of Fish Biology. 2011;78(7):2026-51.

3. White WT, Baje L, Sabub B, Appleyard SA, Pogonoski JJ, Mana RR. Smooth hammerhead shark, *Sphyrna zygaena* (Linnaeus, 1758). In: White WT, Baje L, Sabub B, Appleyard SA, Pogonoski JJ, Mana RR, editors. Sharks and rays of Papua New Guinea ACIAR Monograph No. 189. Canberra: Australian Centre for International Agricultural Research; 2017. p. 190-1.

4. Cailliet GM, Martin LK, Harvey JT, Kusher D, Welden BA, editors. Preliminary studies on the age and growth of blue (*Prionace glauca*), common thresher (*Alopias vulpinus*), and shortfin mako (*Isurus oxyrinchus*) sharks from California waters. Proceedings International Workshop on Age Determination of Oceanic Pelagic Fishes: Tunas, Billfishes, Sharks, ED Prince and LM Pulos, Eds, NOAA Tech Rep NMFS; 1983.

5. Gervelis BJ, Natanson LJ. Age and growth of the common thresher shark in the Western North Atlantic Ocean. Transactions of the American Fisheries Society. 2013;142(6):1535-45.

6. Smith SE, Rasmussen RC, Ramon DA, Cailliet GM. The biology and ecology of thresher sharks (Alopiidae). In: Camhi MD, Pikitch EK, Babcock EA, editors. Sharks of the open ocean: biology, fisheries and conservation. Fish and Aquatic Resources Series. Oxford, Uk: Blackwell Publishing; 2008. p. 60-8.

7. Rigby C. *Maculabatis astra*. The IUCN Red List of Threatened Species. 2016. doi: <http://dx.doi.org/10.2305/IUCN.UK.2016-3.RLTS.T195455A104184896.en>.

8. Jacobsen IP, Bennett MB. Life history of the blackspotted whipray Himantura astra. J Fish Biol. 2011;78(4):1249-68. Epub 2011/04/06. doi: 10.1111/j.1095-8649.2011.02933.x. PubMed PMID: 21463319.

9. Pierce SJ, White WT, Jacobsen IP, Barratt PJ, Last PR, Kyne PM. *Neotrygon picta*. The IUCN Red List of Threatened Species. 2015. doi: <http://dx.doi.org/10.2305/IUCN.UK.2015-4.RLTS.T195464A68636975.en>.

10. Jacobsen IP, Bennett MB. Age and growth of *Neotrygon picta, Neotrygon annotata* and *Neotrygon kuhlii* from north‐east Australia, with notes on their reproductive biology. Journal of Fish Biology. 2010;77(10):2405-22.

11. Dulvy NK, Pardo SA, Simpfendorfer CA, Carlson JK. Diagnosing the dangerous demography of manta rays using life history theory. PeerJ. 2014;2:e400. Epub 2014/06/12. doi: 10.7717/peerj.400.

12. Cuevas-Zimbrón E, Sosa-Nishizaki O, Pérez-Jiménez JC, O’Sullivan JB. An analysis of the feasibility of using caudal vertebrae for ageing the spinetail devilray, *Mobula japanica* (Müller and Henle, 1841). Environmental Biology of Fishes. 2013;96(8):907-14.

13. Walls RHL, Pardo SA, Bigman JS, Clark TB, Smith WD, Bizzarro JJ. *Mobula thurstoni*. The IUCN Red List of Threatened Species. 2016. doi: <http://dx.doi.org/10.2305/IUCN.UK.2016-1.RLTS.T60200A3091468.en>.

14. Last PR, Naylor GJ, Séret B, White WT, de Carvalho M, Stehmann M. Rays of the World. Melbourne, Australia: CSIRO Publishing; 2016.

15. Seck AA, Diatta Y, Diop M, Guelorget O, Reynaud C, Capapé C. Observations on the reproductive biology of the blackchin guitarfish *Rhinobatos cemiculus* E. Geoffroy Saint-Hilaire, 1817 (Chondrichthyes, Rhinobatidae) from the coast of Senegal (Eastern Tropical Atlantic). Scientia Gerundensis. 2004;27:19-30.

16. Ali M, Saad A, Kurbaj H. Reproductive cycle and size at sexual maturity of Chondrichthyan fish *Rhinobatos cemiculus* (Rhinobatidae) of the Syrian marine waters. Annals of Agricultural Sciences. 2008;46:21-30.

17. Capapé C, Zaouali J. Distribution and reproductive biology of the blackchin guitarfish, *Rhinobatos cemiculus* (Pisces: Rhinobatidae), in Tunisian waters (Central Mediterranean). Marine and Freshwater Research. 1994;45(4):551-61.

18. Enajjar S, Bradai MN, Bouain A. Age, growth and sexual maturity of the blackchin guitarfish *Rhinobatos cemiculus* in the Gulf of Gabès (southern Tunisia, central Mediterranean). Cahiers de Biologie Marine. 2012;53(1):17.

19. White WT, Dharmadi. Species and size compositions and reproductive biology of rays (Chondrichthyes, Batoidea) caught in target and non-target fisheries in eastern Indonesia. Journal of Fish Biology. 2007;70(6):1809-37. doi: 10.1111/j.1095-8649.2007.01458.x.

20. White J, Simpfendorfer CA, Tobin AJ, Heupel MR. Age and growth parameters of shark-like batoids. Journal of Fish Biology. 2014;84(5):1340-53. Epub 2014/04/08. doi: 10.1111/jfb.12359. PubMed PMID: 24702252.

21. Dulvy NK, Davidson LNK, Kyne PM, Simpfendorfer CA, Harrison LR, Carlson JK, et al. Ghosts of the coast: global extinction risk and conservation of sawfishes. Aquatic Conservation: Marine and Freshwater Ecosystems. 2016;26(1):134-53. doi: 10.1002/aqc.2525.

22. Rossouw GJ. Age and growth of the sand shark, *Rhinobatos annulatus,* in Algoa Bay, South Africa. Journal of Fish Biology. 1984;25(2):213-22.

23. Casselberry GA, Carlson JK. Endangered Species Act Status Review of the Brazilian Guitarfish (*Rhinobatos horkelii*). Report to the National Marine Fisheries Service, Office of Protected Resources SFD Contribution PCB. 2015;15-08.

24. Márquez-Farías JF. Reproductive biology of shovelnose guitarfish *Rhinobatos productus* from the eastern Gulf of California México. Marine Biology. 2007;151(4):1445-54. doi: 10.1007/s00227-006-0599-3.

25. Timmons M, Bray R. Age, growth, and sexual maturity of shovelnose guitarfish, *Rhinobatos productus* (Ayres). Oceanographic Literature Review. 1998;1(45):148.

26. Downton-Hoffman C. Biología del pez guitarra *Rhinobatos productus* (Ayres, 1856) Baja California Sur, México. La Paz, México: CICIMAR-IPN; 2007.

27. Newell B. Status Review Report of Two Species of Guitarfish: *Rhinobatos rhinobatos* and *Rhinobatos cemiculus*. Report to National Marine Fisheries Service, Office of Protected Resources, National Oceanic and Atmospheric Administration.1-68.

28. Başusta N, Demirhan SA, Çiçek E, Başusta A, Kuleli T. Age and growth of the common guitarfish, *Rhinobatos rhinobatos,* in Iskenderun Bay (north-eastern Mediterranean, Turkey). Journal of the Marine Biological Association of the UK. 2008;88(04). doi: 10.1017/s0025315408001124.

29. Abdel-Aziz SH, Khalil AN, Abdel-Maguid SA. Reprodutive cycle of the Rhinobatos rhinobatos in Alexandria Waters, Mediterranean Sea. Aust J Mar Freshwater Res. 1993;44(507-517).

30. Ismen A, Yıgın C, Ismen P. Age, growth, reproductive biology and feed of the common guitarfish (*Rhinobatos rhinobatos* Linnaeus, 1758) in İskenderun Bay, the eastern Mediterranean Sea. Fisheries Research. 2007;84(2):263-9. doi: 10.1016/j.fishres.2006.12.002.

31. Lteif M, Mouawad R, Jemaa S, Khalaf G, Lenfant P, Verdoit-Jarraya M. The length-weight relationships of three sharks and five batoids in the Lebanese marine waters, eastern Mediterranean. The Egyptian Journal of Aquatic Research. 2016;42(4):475-7. doi: 10.1016/j.ejar.2016.09.008.

32. Lteif M, Mouawad R, Khalaf G, Lenfant P, Verdoit-Jarraya M. Population biology of an endangered species: the common guitarfish *Rhinobatos rhinobatos* in Lebanese marine waters of the eastern Mediterranean Sea. Journal of Fish Biology. 2016;88(4):1441-59. Epub 2016/03/02. doi: 10.1111/jfb.12921. PubMed PMID: 26928654.
